# High speed photo-mediated ultrasound therapy integrated with OCTA

**DOI:** 10.1038/s41598-022-23188-8

**Published:** 2022-11-19

**Authors:** Yan Li, Yuchen Song, Runze Li, Wangcun Jia, Fengyi Zhang, Xiaoming Hu, Lidek Chou, Qifa Zhou, Zhongping Chen

**Affiliations:** 1 Beckman Laser Institute, University of California, Irvine, Irvine, CA 92612 USA; 2 Department of Biomedical Engineering, University of California, Irvine, Irvine, CA 92617 USA; 3 Department of Ophthalmology and Biomedical Engineering, University of Southern California, Los Angeles, CA 90089 USA

**Keywords:** Optical techniques, Optics and photonics, Biophotonics, Biomedical engineering

## Abstract

Photo-mediated Ultrasound Therapy (PUT), as a new anti-vascular technique, can promote cavitation activity to selectively destruct blood vessels with a significantly lower amount of energy when compared to energy level required by other laser and ultrasound treatment therapies individually. Here, we report the development of a high speed PUT system based on a 50-kHz pulsed laser to achieve faster treatment, decreasing the treatment time by a factor of 20. Furthermore, we integrated it with optical coherence tomography angiography (OCTA) for real time monitoring. The feasibility of the proposed OCTA-guided PUT was validated through in vivo rabbit experiments. The addition of OCTA to PUT allows for quantitative prescreening and real time monitoring of treatment response, thereby enabling implementation of individualized treatment strategies.

## Introduction

Port-Wine Stain (PWS) is characterized by ectatic capillaries and postcapillary venules located predominantly in the papillary and mid-reticular layers of the dermis^1^. Studies have recorded that PWS has an incidence of about 3–5 cases per 1000 newborn babies and grows commensurately with the affected individual^2^. Since the early 1980s, Pulsed Dye Laser (PDL) has been considered to be the gold standard for treatment of PWS^3^, which provides improvement through selective destruction of vasculature. However, very small and deep vessels are less likely to respond and a reported 20–30% of PWS show resistance to PDL^4^. To further improve treatment outcomes, a considerable number of vascular-selective lasers with different wavelengths have been employed. For example, Nd:YAG lasers at 532 nm are commonly used for PDL treatment-resistant PWS. This laser has a limited penetration depth and is primarily used for treating superficial vascular lesions. Moreover, some studies reported that Nd:YAG laser based treatment has a slightly reduced efficacy when compared with PDL and has an increased occurrence of side effects^5^. Nd:YAG laser at 1064 nm has the highest depth of penetration and the lowest epidermal melanin absorption. However, its adverse effects (such as pigmentary change and scarring) are more frequent because higher fluence is generally required^6^. Photodynamic therapy (PDT) is an advancing treatment option, and PDT with efficacy equivalent to or possibly superior than PDL has been reported^7^. However, this technique introduces a chemical photosensitizer and requires the avoidance of sun exposure for days to weeks.

Despite advancements in laser treatment, complete PWS resolution remains rare. It was reported that less than 20% PWS can be completely cleared^8^ and approximately half of all PWS patients bear lesions that are recalcitrant to current treatment options^9^. One main cause is the limited laser fluence: laser fluence needs to be high enough to achieve therapeutic effectiveness while maintaining within the ANSI safety limits to avoid damaging the surrounding tissue^3^. Another cause is the insufficient preoperative assessment of PWS lesions and the lack of a real time monitoring tool during treatment. Because each patient responds to the treatment differently depending on the initial lesion conditions, even with the same treatment settings, PWS patients may receive either too much energy resulting in scarring and burn injuries, or insufficient laser energy leading to ineffective treatment. Most diagnosis and clinical outcome measurement of PWS treatments rely on visual assessments, which are susceptible to inter- and intra-observer variations and provide limited information. Therefore, it is imperative to develop a novel therapeutic technique with improved safety and efficacy, as well as a non-invasive diagnostic and monitoring tool which can provide cross-sectional tissue anatomy and physiology for valid, reliable outcome measurement.

Photo-mediated Ultrasound Therapy (PUT)^10–12^, a new anti-vascular technique based on photospallation, applies nanosecond laser pulses and ultrasound bursts simultaneously to promote cavitation activity to destruct blood vessels. PUT is highly selective and provides a high-precision localized treatment because the cavitation will be limited to the blood vessels. Such localization can be achieved because hemoglobin has greater capacity for absorption at working wavelength when compared to surrounding tissues. More importantly, PUT can avoid unwanted damage to the surrounding tissues because it uses much lower energy, for both laser and ultrasound, than individually required by traditional laser treatments and traditional therapeutic ultrasound^13^. Therefore, PUT holds a great potential to be a new therapeutic method for PWS with improved safety and efficacy.

To visualize vasculature change during port-wine stain treatment, several non-invasive methods have been applied, such as ultrasound (US) imaging, optical coherence tomography (OCT)^14–17^, and photoacoustic imaging. Ultrasound imaging has been investigated to study hemodynamics of port-wine stain^18^, however its limitation resolution (~ 100 µm) limits the accuracy. Photoacoustic imaging, an emerging imaging modality, is able to map vasculature with high resolution of ~ 10 µm, and been reported to quantify vessel diameter, depth, and density^19^. However, detailed layered architecture of skin tissue is lacking. OCT is promising non-invasive tool for investigating skin morphology, can provide near histopathological information before and during the course of treatment. A number of extensions of OCT capabilities for functional imaging of tissue physiology have been developed, including Doppler OCT, OCT angiography (OCTA), and optical coherence elastography^20–27^. Of these, OCTA is capable of generating high-resolution tomographic images of vasculature and has been used for pre-treatment assessment and treatment monitoring during the laser therapy^28,29^, where it has demonstrated strong capability of OCTA to evaluate treatment outcome. Both OCT and OCTA have had a growing impact in the field of dermatology.

In this paper, we reported the development of a high-speed OCTA-guided PUT system that uses a 50-kHz pulsed laser to achieve faster treatment. In vivo rabbit experiments were performed to test the feasibility of this proposed technology. We demonstrated a reduction in treatment time by a factor of 20.

## Results

**Figure 1:**
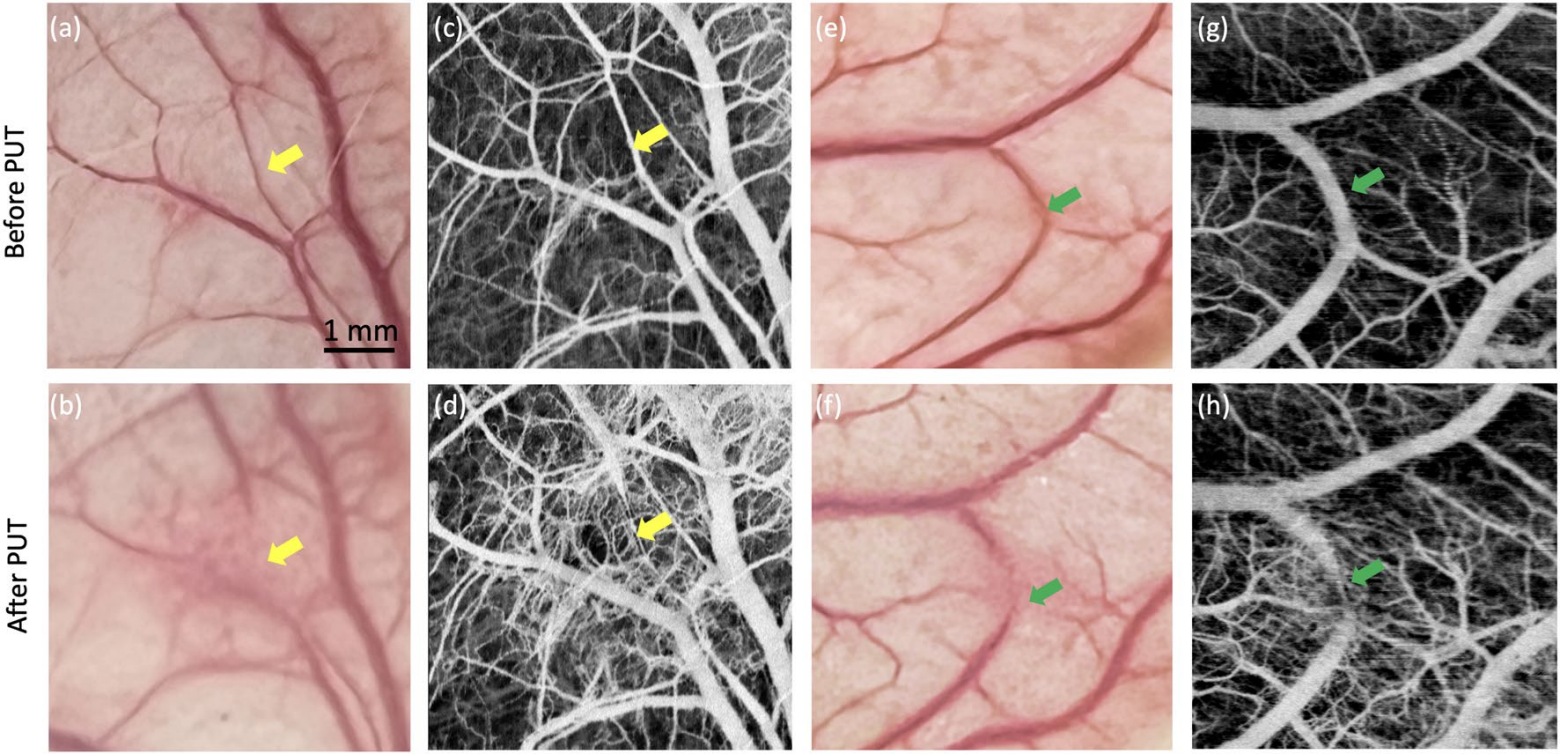
Photo and OCTA images of rabbit ear. (**a**,**b**) Photo of rabbit ear before and after PUT. (**c**,**d**) OCTA images before and after PUT. (**e**,**f**) Photo of rabbit ear before and after PUT. (**g**,**h**) OCTA images before and after PUT.

Figure 1 shows the representative OCTA images from two sites from the ears of rabbits. Figure 1a,c show a photo and OCTA image of the rabbit ear before PUT treatment, where the treatment area is labeled by yellow arrows. Figure 1d shows an OCTA image of the rabbit’s ear after PUT treatment, where a blood vessel destruction can be clearly visualized as indicated by the yellow arrow. The diameters of treated blood vessel before and after treatment were 83 µm and 0 µm, respectively. This is consistent with the corresponding photo in Fig. 1b. Figure 1e–h show the photos and OCTA images of another area on the rabbit ear, where the narrowing of a blood vessel can be identified. This is indicated by the green arrows. The diameters of treated blood vessel before and after treatment were 330 µm and 67 µm, respectively. From the photos of blood vessels alone, it is difficult to determine if the treatment results in vessel destruction or vessel narrowing, whereas OCTA provides a much clearer picture and more accurate outcome measurement.

## Discussion and conclusion

An OCTA-guided PUT system with high-speed nanosecond laser was demonstrated and its feasibility was validated via in vivo rabbit experiments. In reported studies, most PUT systems apply a 10 Hz pulsed laser, which inhibits fast treatment^10–12^. To address this issue, our PUT system features a high-speed pulsed laser that reduces the treatment time by a factor of 20. Furthermore, the addition of OCTA provides a non-invasive diagnostic and monitoring tool and has the potential to further improve the safety and efficiency of PUT.

**Figure 2:**
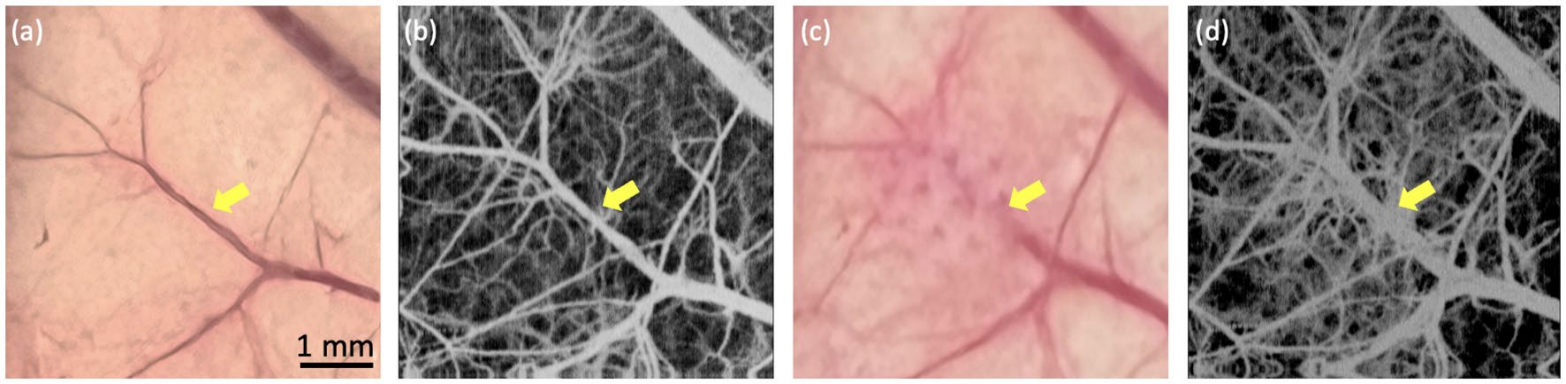
Photo and OCTA images of rabbit ear. (**a**,**b**) Photo and OCTA image of rabbit ear before PUT. (**c**,**d**) Photo and OCTA image after PUT.

While the proposed OCTA-guided PUT system is a promising tool for PWS, a few challenges still need be addressed to successfully translate this technology for clinical applications. In terms of technical improvement, a ring-shaped high intensity focused ultrasound (HIFU) transducer is necessary because the sample has to be placed between the laser beam and the acoustic beam in current setup, which limits the thickness of the imaging sample. Additionally, Fourier domain mode locking (FDML) lasers with higher sweep rates can be incorporated to further decrease OCTA imaging time. For animal studies, a larger sample size is necessary to establish a histopathologic baseline to determine initial treatment. In our study, we found that even with the same treatment settings, the outcomes vary greatly because treatment response is highly associated with initial conditions of a lesion. As shown in Fig. 1h, the blood vessel with a larger diameter wasn’t fully destructed and instead narrowing was observed. Figure 2 shows another example where a blood vessel at a different depth dilated a little bit after treatment with the same treatment parameters. Also, extra blood capillary can be seen around the target vessel because the local stimulation caused perfusion increase after PUT therapy^30^. For the next step, we will investigate the optimal system parameters, including laser wavelength, laser fluence, HIFU pressure, HIFU duty cycle, time delay between laser and ultrasound, and treatment duration; eventually correlate them with the initial lesion condition. In addition, although no obvious thermal effect was observed except minor redness, we will add temperature and birefringence measurements in our future study to provide an accurate evaluation of side effects.

In summary, we reported the first OCTA-guided PUT system with a significantly shorter treatment time. Our technique has been validated in an in vivo rabbit model. We believe that with further improvements, the proposed technology has the capability to enhance clinicians’ ability to measure treatment outcome and provide insight into the optimal treatment dose and duration, thus enabling individualized patient management.

## Materials and methods

**Figure 3:**
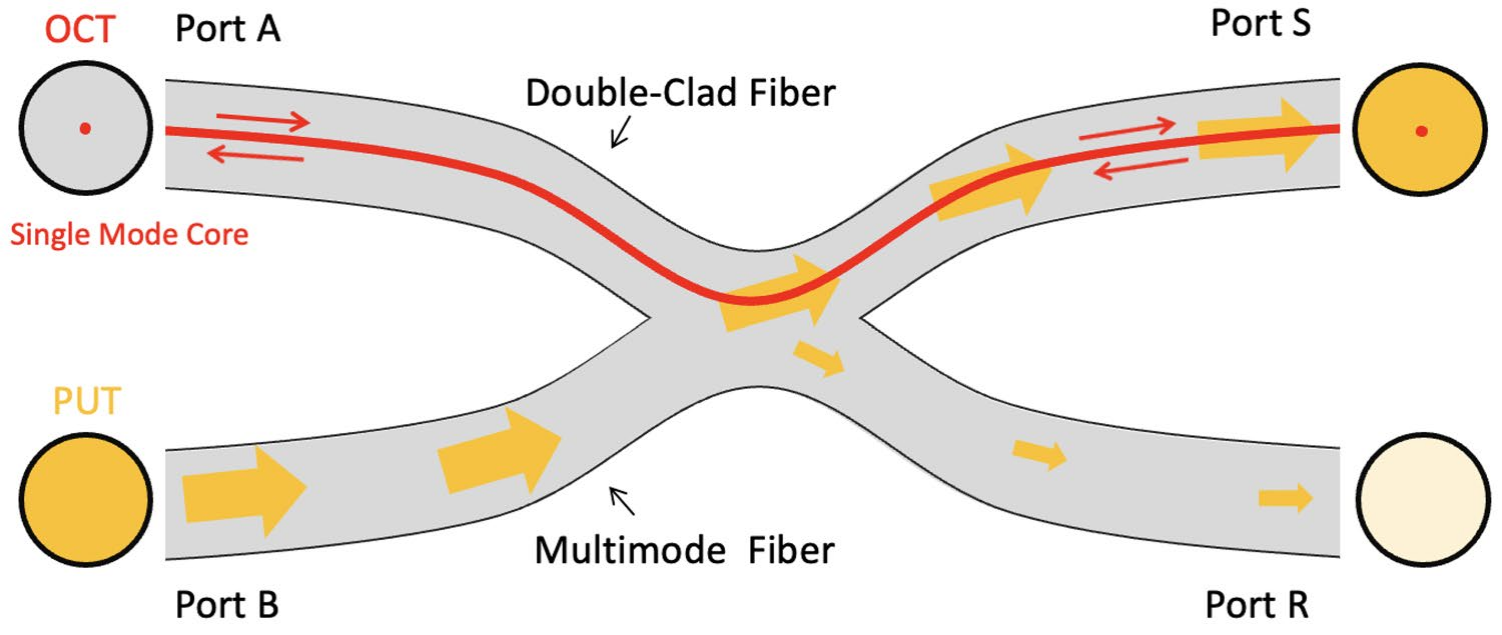
Diagram of Double-clad fiber coupler.

To fully combine OCT with PUT, a double-clad fiber (DCF) was used to transmit combined beam where OCT light propagates in the single mode core and the laser pulses propagates in the multimode inner cladding. DCF is necessary because OCT requires single mode propagation to maintain coherence, and laser pulse requires a large mode area for delivering high power laser pulses. Therefore, we collaborated with Castor Optics to design and manufacture a double-clad fiber (DCF) coupler, as shown in Fig. 3. The 2 × 2 DCF coupler combines a DCF (single mode core Ø 9 µm with 0.12 NA, surrounded by a multimode inner cladding Ø 105 µm with 0.2 NA.) with a standard step-index multimode fiber (MMF, multimode core Ø 105 µm with 0.15 NA). Light in the single mode core, which is used for OCT/OCTA imaging, can transmit over the 1250–1550 nm wavelength range with a maximum insertion loss of 0.5 dB. Light in the multimode regions, which is used for laser pulse delivery in PUT, propagates from Port B to Port S over a wider wavelength range of 400–1600 nm with an efficiency larger than 75%.

**Figure 4:**
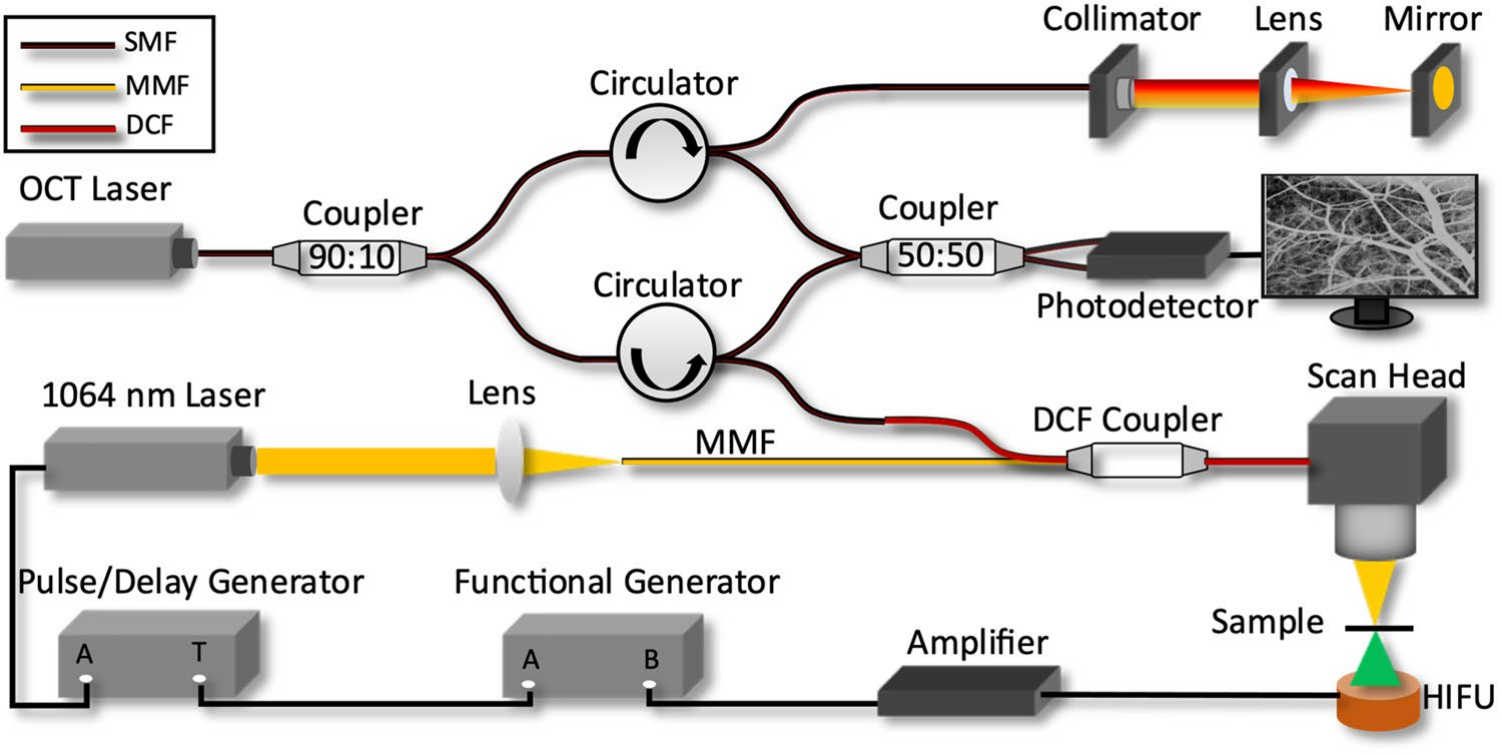
Schematic of OCTA-guided PUT system. *DCF* double clad fiber, *SMF* single mode fiber, *HIFU* high intensity focused ultrasound, *MMF* multimode fiber.

**Figure 5:**
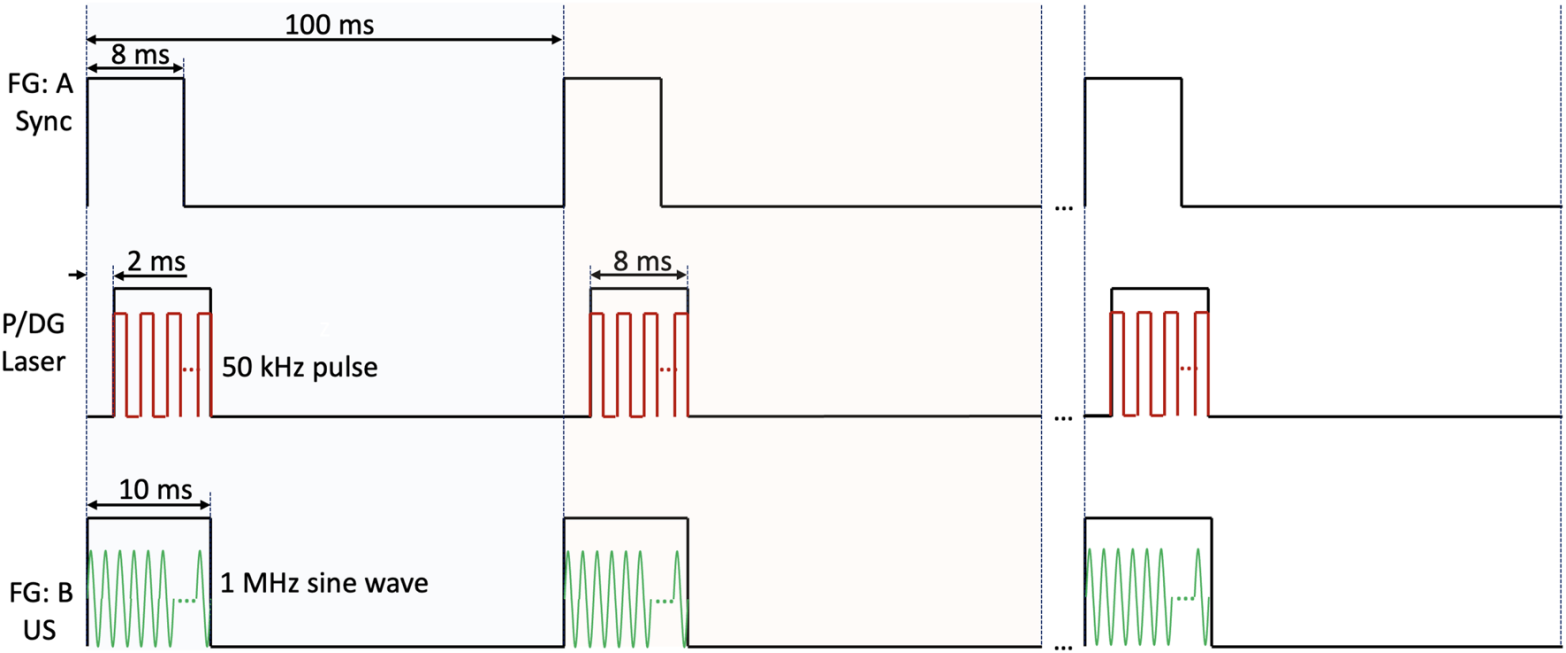
Timing diagram. *FG* function generator, *P/DG* pulse delay generator, *US* ultrasound.

The schematic diagram of the OCTA-guided PUT system is shown in Fig. 4. The DCF coupler is applied to combine OCT illumination and PUT laser pulses. OCT and PUT share the same optical path in the scan head, which consists of a collimator, a high-speed 2-axis galvo scanner, and a scan lens. OCT uses a 1310 nm Micro-Electro-Mechanical (MEMS)-tunable Vertical Cavity Surface Emitting Laser (VCSEL) swept source with a sweep rate of 100 kHz and a bandwidth of 100 nm. The sensitivity, axial resolution, and lateral resolution of our SSOCT is ~ 110 dB, 9 µm, and 30 µm, respectively. For PUT, a 1064-nm nanosecond laser with a repetition rate of up to 100 kHz is utilized. The output of the nanosecond laser is focused by a condenser lens into the multimode fiber (MMF). A function generator is used to synchronize the pulse/delay generator for pulsed laser emission as well as generating a sine wave used for ultrasound emission. Channel A of the function generator outputs a square wave with a repetition rate of 10 Hz and a duty cycle of 8%. Through the pulse/delay generator, 50-kHz pulses, gated by the aforementioned square wave with 2 ms delay, are produced to control and synchronize laser emissions. Channel B of the function generator produces a 1 MHz sine wave gated by a 10 Hz square wave with 10% duty cycle. Two output signals from the function generator are synchronized. The gated sine wave is power amplified to drive HIFU transducer with a center frequency of 1 MHz. Figure 5 shows a detailed timing diagram for the OCTA-guided PUT system. The HIFU transducer, coaxially aligned with the laser beam, is immersed in warm water (~ 37 °C) for acoustic coupling.

We performed in vivo experiments on the ears of rabbits. Each rabbit (male, New Zealand white rabbit) was administrated a ketamine-xylazine mixture (35 mg/kg and 5 mg/kg, respectively) for initial anesthesia. After conforming to the proper depth of anesthesia, a rabbit ear was shaved and secured on a ring-shaped holder for OCTA-guided PUT. Each site was treated with a laser fluence of ~ 2 mJ/cm^2^ and a peak negative ultrasound pressure of ~ 0.5 MPa for a total time of 30 s. In our study, three sites from three rabbits were imaged in total. During the treatment, the aiming beam (red light) from nanosecond laser was used to select the ROI based direct visualization. Since aiming beam and OCT share the same optical path, the ROI is normally the center of our OCTA images.

**Figure 6:**
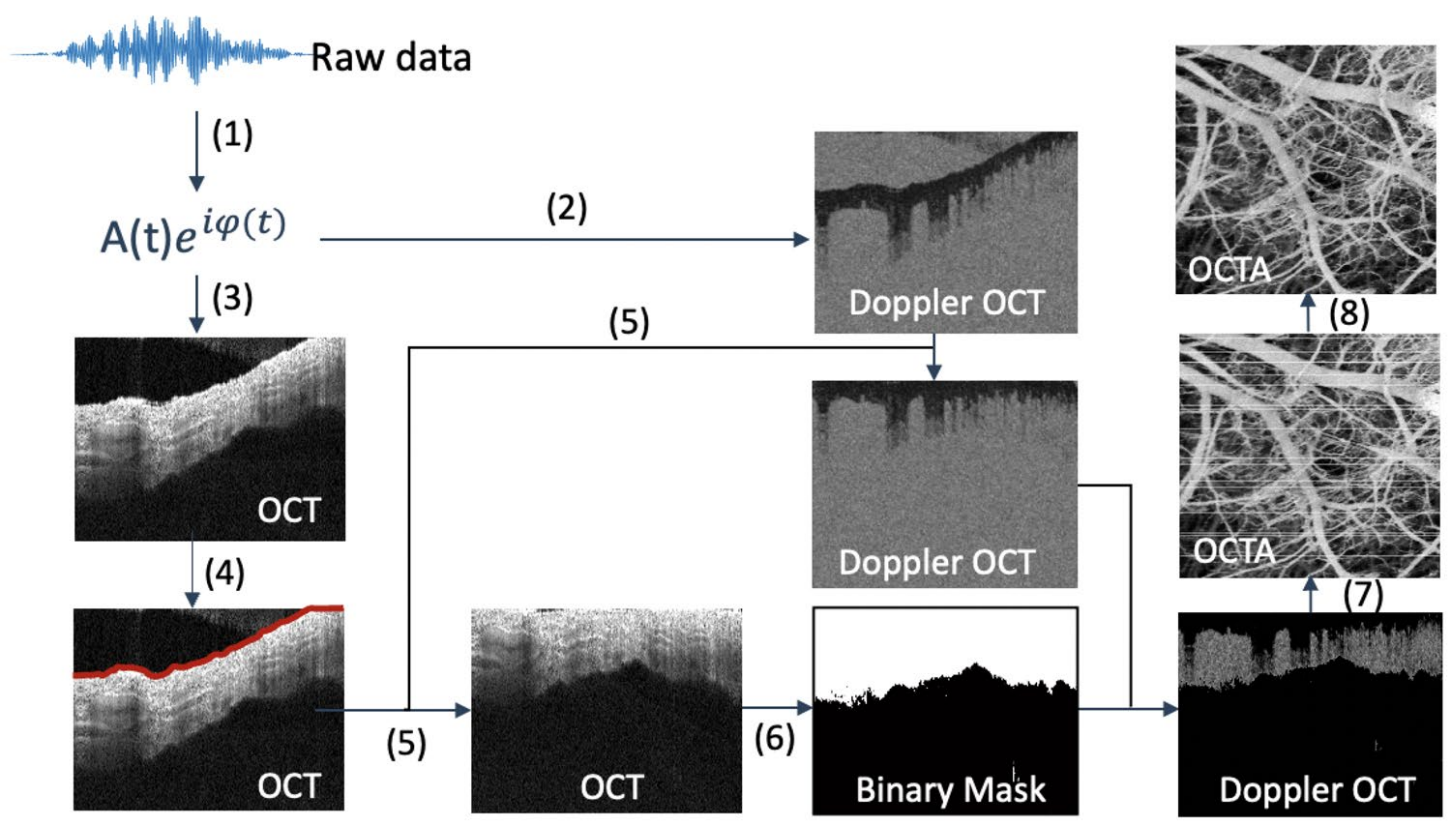
Flow chart of OCTA algorithm. (1) Hanning window and FFT. (2) Intensity-based Doppler variance method to extract blood vessel. (3) OCT image. (4) Edge detection based on OCT image. (5) Flatten image target surface. (6) Generate a binary mask to remove background from Doppler image. (7) Scanning. (8) Bulk motion removal using a low pass filter.

For OCTA, we applied an intensity-based Doppler variance algorithm^31^ to capture the fluctuation caused by blood flow, as shown in Fig. 6. OCTA was performed before and right after PUT. An inter-frame scanning protocol is applied where consequent 8 cross-sectional B-scans are acquired at the same position and calculated to map blood vessel, as indicated in step (1). To better visualize vascular network, we flatten the Doppler OCT image surface and removed background based on OCT image, as shown in steps (3–6). With scanning (7), en face vasculature was achieved where many horizontal stripes caused by bulk motion can be found. For further improvement, we processed obtained vascular network with a low pass filter in the step (8) and successfully removed most of the strips.

### Ethics approval

This study is approved by Institutional Review Board (IRB) and the Institutional Biosafety Committee (IBC) of University of California, Irvine (protocol #AUP-19-042). All experiments were carried out in compliance with the ARRIVE guidelines. Studies were performed in accordance with local and national ethical guidelines and regulations.

## Data Availability

The data that support the findings of this study are available from the corresponding author upon reasonable request.
